# Rules for Bleeding in Pneumonia

**Published:** 1852-05

**Authors:** 


From the Edinburgh Monthly Journal.

*Rules for Bleeding in Pneumonia.*

The following judicious remarks by Dr. Bennett, are perfectly in accordance with our own experience:

"If we are called to a case at a very early period, before exudation is poured out, and before dullness as its physical sign is characterized, but when, notwithstanding, there have been rigors, embarrassment of respiration, more or less pain in the side, commencing crepitation, then bleeding will often cut the disease short. This state of matters is rarely seen in public hospitals. When, on the other hand, there is perfect dullness over the lung, increased vocal resonance, and rusty sputum, then exudation blocks up the air-cells, and can only be got rid of by that exudation being transformed into pus, and excreted by the natural passages. In such a case bleeding checks the vital powers necessary for these transformations, and, as a general rule, if the disease be not fatal, will delay the recovery. I believe this to be the cause of so much mortality from pneumonia in hospitals where bleeding is largely practiced, for, in general, individuals affected do not enter until the third or fourth day, when the lung is already hepatized.

